# Acute hematologic toxicity prediction using dosimetric and radiomics features in patients with cervical cancer: does the treatment regimen matter?

**DOI:** 10.3389/fonc.2024.1365897

**Published:** 2024-05-21

**Authors:** Haizhen Yue, Xiaofan Li, Jing You, Pujie Feng, Yi Du, Ruoxi Wang, Hao Wu, Jinsheng Cheng, Kuke Ding, Bin Jing

**Affiliations:** ^1^ National Institute for Radiological Protection, Chinese Center for Disease Control and Prevention, Beijing, China; ^2^ Key Laboratory of Carcinogenesis and Translational Research (Ministry of Education/Beijing), Department of Radiation Oncology, Peking University Cancer Hospital & Institute, Beijing, China; ^3^ Beijing Key Laboratory of Fundamental Research on Biomechanics in Clinical Application, School of Biomedical Engineering, Capital Medical University, Beijing, China; ^4^ Chinese Center for Disease Control and Prevention, Beijing, China

**Keywords:** radiomics, cervical cancer, hematologic toxicity, chemoradiotherapy, bone marrow

## Abstract

**Background:**

Acute hematologic toxicity (HT) is a prevalent adverse tissue reaction observed in cervical cancer patients undergoing chemoradiotherapy (CRT), which may lead to various negative effects such as compromised therapeutic efficacy and prolonged treatment duration. Accurate prediction of HT occurrence prior to CRT remains challenging.

**Methods:**

A discovery dataset comprising 478 continuous cervical cancer patients (140 HT patients) and a validation dataset consisting of 205 patients (52 HT patients) were retrospectively enrolled. Both datasets were categorized into the CRT group and radiotherapy (RT)-alone group based on the treatment regimen, i.e., whether chemotherapy was administered within the focused RT duration. Radiomics features were derived by contouring three regions of interest (ROIs)—bone marrow (BM), femoral head (FH), and clinical target volume (CTV)—on the treatment planning CT images before RT. A comprehensive model combining the radiomics features as well as the demographic, clinical, and dosimetric features was constructed to classify patients exhibiting acute HT symptoms in the CRT group, RT group, and combination group. Furthermore, the time-to-event analysis of the discriminative ROI was performed on all patients with acute HT to understand the HT temporal progression.

**Results:**

Among three ROIs, BM exhibited the best performance in classifying acute HT, which was verified across all patient groups in both discovery and validation datasets. Among different patient groups in the discovery dataset, acute HT was more precisely predicted in the CRT group [area under the curve (AUC) = 0.779, 95% CI: 0.657–0.874] than that in the RT-alone (AUC = 0.686, 95% CI: 0.529–0.817) or combination group (AUC = 0.748, 95% CI: 0.655–0.827). The predictive results in the validation dataset similarly coincided with those in the discovery dataset: CRT group (AUC = 0.802, 95% CI: 0.669–0.914), RT-alone group (AUC = 0.737, 95% CI: 0.612–0.862), and combination group (AUC = 0.793, 95% CI: 0.713–0.874). In addition, distinct feature sets were adopted for different patient groups. Moreover, the predicted HT risk of BM was also indicative of the HT temporal progression.

**Conclusions:**

HT prediction in cervical patients is dependent on both the treatment regimen and ROI selection, and BM is closely related to the occurrence and progression of HT, especially for CRT patients.

## 1 Introduction

Cervical cancer is the most common gynecological malignancy with approximately 604,100 new cases and 341,800 deaths estimated in 2020 worldwide (1). For patients diagnosed with locally advanced cervical cancer (LACC), concurrent chemoradiotherapy (CRT) is the established standard treatment strategy (2, 3). However, most patients undergoing CRT will experience acute hematologic toxicity (HT), which may compromise their tolerance to subsequent treatment, leading to unplanned interruptions of CRT. This poses a threat to tumor control and increases the risk of complications (4–8). Therefore, early and accurate prediction of the risk for acute HT may be essential to improve the clinical outcome of cervical cancer.

Previous studies have demonstrated a correlation between HT occurrence during CRT and the dosimetric factors of pelvic bone marrow (PBM), such as the V5, V10, V20, and V30 (9–14), which can be employed as the predictors of acute bone marrow suppression. Furthermore, a systematic review by Corbeau Anouk et al. (15) revealed that the V10 (>95%–75%), V20 (>80%–65%), and V40 (>37%–28%) of PBM were significantly associated with HT in LACC patients receiving cisplatin-based CRT. The literature also raised concerns about the correlation between pathological features and HT of cervical cancer (16); however, this study only identified positive HT as grade 2 or higher. These findings indicated that dosimetric factors are related to adverse events (e.g., HT) during CRT, which can be combined with demographic and clinical (e.g., pathology) characteristics to predict the occurrence of HT. However, these features struggle to reflect the individual differences among patients, thus making the individualized prediction of HT ineffective.Radiomics is a quantitative imaging technique that has been extensively employed in personalized disease diagnosis and prognosis prediction (17–19). Radiomics depicts both macroscopic and microscopic characteristics for selected regions of interest (ROIs) on the image and has shown certain relationships with genomic, pathologic, and histologic information (20). Previous studies have confirmed that combining dosimetric and clinical features with radiomics could predict HT in cervical cancer patients (8, 21). However, these studies suffer from limited sample size, potentially leading to relatively low predictive performance. Additionally, an overlooked latent factor in these studies is the mixing of patients who underwent different combination treatment regimens for HT prediction. For instance, some patients received CRT, while others underwent radiotherapy (RT) alone within the focused RT duration. CRT and RT alone may induce different treatment responses in patients, which will potentially affect HT occurrence and prediction. However, no study has systematically explored the discrepancies in HT prediction among cervical cancer patients with different treatment regimens within the focused RT duration, which was defined as the interval spanning from 1 week prior to the initiation of radiotherapy to 1 week after its completion.

In this study, we would fill the gap by utilizing a large sample size of cervical cancer patients for HT prediction. Three comprehensive models were constructed for patients with CRT, RT alone, and their combination using the integrated clinical, dosimetric, and radiomics features from three clinically relevant ROIs in planning CT images. The most discriminative ROIs and the related contributing feature sets were confirmed for each model and compared with each other. Subsequently, the time-to-event analysis was conducted using the predictive ROIs to understand the temporal progression for risky patients with different treatment plans. This comprehensive study could help to further understand the HT occurrence probability for cervical cancer patients within the focused RT duration, which may potentially guide the development of individualized treatment regimens and improve the clinical treatment outcome. We infer that patients with different treatment regimens may display unlike HT prediction performance and contributing features.

## 2 Materials and methods

### 2.1 Patient enrollment

#### 2.1.1 Discovery dataset

A large cohort of 574 patients diagnosed with 2018 International Federation of Gynecology and Obstetrics (FIGO) stage IA–IVB (22) cervical cancer treated at Beijing Cancer Hospital between September 2010 and February 2021 was included in this retrospective study. Inclusion criteria comprised of patients who had pathologically proven cervical carcinoma, no evidence of distant metastasis based on CT or PET, and no history of renal, hepatic, hematologic disease, or other systemic diseases; and complete blood count (CBC) data within 1 week before the start of radiation and weekly CBC during the treatment period. Patients were excluded if they 1) had a history of pelvic radiotherapy or systemic chemotherapy; had 2) long-term severe anemia before radiotherapy; 3) had inconsistent prescription doses, incomplete blood data, or severe radiation interruptions or had not completed the full course of radiotherapy; and 4) had a history of prior malignancies or recurrent tumors.

This study exclusively focused on the occurrence of HT within the RT duration. To avoid the potential influence of pre-RT chemotherapeutic agents on HT results, patients without chemotherapy within 1 month prior to and 1 week following the completion of RT were categorized as the RT-alone group. Patients who received more than one cycle of chemotherapy within the RT duration were defined as the CRT group. The study included 478 patients who met the aforementioned criteria, out of which 140 patients had acute HT (grade ≥ 3), which was characterized by a white blood cell count below 1.9 × 10^9^/L, neutrophil count below 0.9 × 10^9^/L, platelet count below 49 × 10^9^/L, or hemoglobin below 79 × 10^9^/L. According to the treatment regimen, 223 and 255 patients were divided into the RT-alone group and the CRT group, respectively. **Table 1** provides an overview of patients’ demographical and clinical characteristics. Our study was approved by the ethics committee of Beijing Cancer Hospital.

**Table 1:** The clinical characteristics of all patients.

Clinical factors	Discovery dataset	Validation dataset
**Age** (median, mean ± SD, range)	59, 57.7 ± 10.0, 25–79	59, 57.8 ± 10.2, 33–77
**Number**	478	205
Pathological type		
Squamous carcinoma	432	176
Adenocarcinoma	31	20
Adenosquamous carcinoma	5	5
Others	10	4
Chemotherapy		
Yes	255	118
No	223	87
FIGO stage (n, %)		
I	123	101
II	221	83
III	112	18
IV	22	2
Acute hematotoxicity		
**≥3**	140	52
**<3**	338	153
Radiotherapy prescription		
45/60 Gy/25 fractions	478	0
50 Gy/25 fractions	0	205
Concurrent chemotherapy		
Weekly CDDP	167	71
Triweekly CDDP	58	47
None	223	87

FIGO, International Federation of Gynecology and Obstetrics; SD, standard deviation; CDDP, cis-diamminedichloroplatinum.

#### 2.1.2 Validation dataset

**Figure 1:**
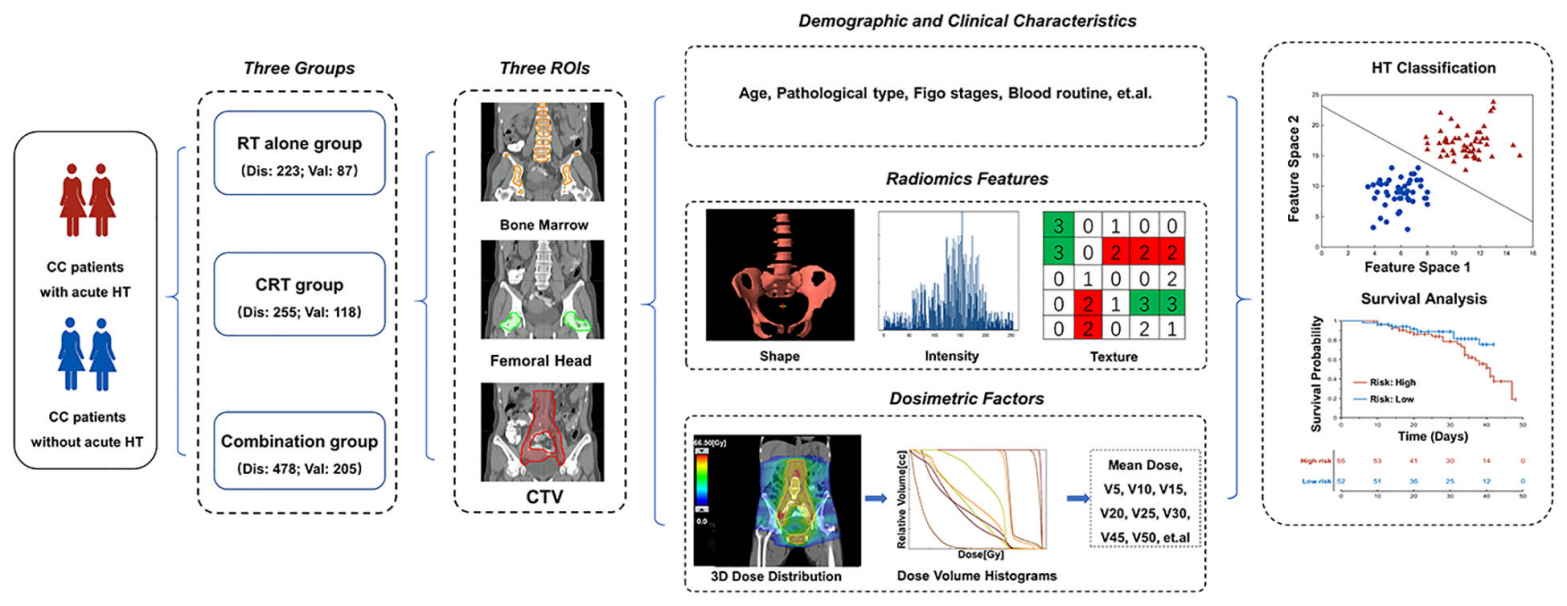
The workflow of the study. CC, cervical cancer; RT, radiotherapy; CRT, chemoradiotherapy; HT, hematologic toxicity; FIGO, International Federation of Gynecology and Obstetrics; Dis, discovery dataset; Val, validation; CTV, clinical target volume.

To validate the generalization of the acute HT prediction model, a total of 356 cervical cancer patients with FIGO stage IA–IVB who received postoperative radiotherapy at our department from January 2015 to June 2016 were collected. The exclusion and inclusion criteria were the same as those for the discovery dataset. Ultimately, a total of 205 patients (52 acute HT patients) meeting the inclusion/exclusion criteria were finally selected as validation dataset, and 118 and 87 patients were classified into the CRT and RT-alone groups, respectively. **Table 1** summarizes the demographical and clinical characteristics of these patients. The whole pipeline of the paper is illustrated in **Figure 1**.

### 2.2 CT Simulation for radiotherapy

The simulation and irradiation procedures were conducted with the patient in a supine position and with a relatively full bladder, which was achieved by emptying the bowels and bladder 1 hour before the simulation and then administering 500 mL of water. The abdominopelvic CT was obtained using a Siemens SOMATOM Sensation Open CT scanner (Siemens, Erlangen, Germany), with a continuous slice thickness of 5 mm, a resolution of 512 × 512, and uniform pixel size of 1.3 mm × 1.3 mm.

### 2.3 ROI definition in planning CT

This study utilized rigorous methodology to select three treatment planning ROIs for subsequent analysis, which included the clinical target volume (CTV), femoral head (FH), and bone marrow (BM). To ensure the consistency and robustness of ROI segmentation, according to the Radiation Therapy Oncology Group (RTOG) guidelines (23–26), all ROIs were initially segmented using a deep learning-based automatic segmentation software, followed by subsequent meticulous review and correction conducted by experienced clinicians (X.G. and J.Y.) and additionally confirmed by a senior (more than 15 years’ experience) physician (X.L.) on the Eclipse treatment planning system (Eclipse, Varian Medical Systems, Palo Alto, CA, USA). Specifically, the pelvic bone marrow was defined as the entire bony structure within the range of potential exposure, encompassing the marrow volume of the ilium, pubis, ischium, and sacrum and the BM volume upper bounded by the 12th thoracic to the fifth lumbar vertebrae. Specifically, the spatial definition criteria for three ROIs in both validation and discovery datasets were kept the same.

### 2.4 RT treatment regimen

All enrolled patients underwent external beam radiation therapy (EBRT) utilizing intensity-modulated radiation therapy (IMRT) or volumetric modulated arc therapy (VMAT), which was generated using the Eclipse treatment planning system (V.13.6 or V.15.6). In addition, the treatment regimen of CRT was determined in accordance with the guidelines established by the National Comprehensive Cancer Network (NCCN) (27).

For the discovery dataset, EBRT was administered with two dose levels simultaneously: 60.0 Gy and 45.0 Gy to lymph node gross tumor volume (GTVnd) and CTV, respectively, 25 fractions, and 10-MV photon, a single fraction per day. In the validation dataset, patients received EBRT with a prescribed dose of 50.0 Gy to CTV at 25 daily fractions. Moreover, a portion of patients received an intrauterine brachytherapy boost utilizing the iridium-192 approach in 3 to 5 weeks after the first fraction of the EBRT. Notably, the equivalent dose in 2 Gy (EQD2), calculated under the assumption of an α/β ratio of 10, was maintained within a range of 80 Gy to 85 Gy, referencing point A. The biologically effective dose was calculated considering both internal and external exposures, as well as the treatment interruption caused by acute HT (28, 29).

### 2.5 Dosimetric factors

Two types of dosimetric factors were extracted, namely, mean dose and volume-based metrics such as Vx, where Vx represents the tissue receiving × Gy or above. In order to comprehensively assess the influence of dosimetry on the occurrence of HT, multiple ROIs were collected including BM, FH, and CTV. Within the BM region, three detailed subregions were further analyzed: iliac marrow (IM), sacral marrow (SM), and vertebral marrow (VM). Specifically, the mean dose (14, 30) and V5, V10, V15, V20, V25, V30, V35, V40, V45, and V50 were extracted from the BM, IM, SM, and VM. For the FH region, mean dose and V10, V20, V25, V30, and V35 were considered. Notably, only the mean dose was considered for CTV. To facilitate ROI volume and dosimetric factor extraction, C#-based scripts were developed using the Eclipse Scripting Application Programming Interface (ESAPI) research mode provided by the Eclipse Treatment Planning System (TPS) version 15.6.

### 2.6 Chemotherapy

The concurrent chemotherapy consisted of two main regimens: 1) weekly single-agent cisplatin repeated at 7-day intervals for four to six cycles or 2) triweekly combination regimen repeated at 21-day intervals for one to three cycles. All patients underwent a blood routine examination before each cycle of chemotherapy. The chemotherapy was postponed if the neutrophil count was less than 1.5 × 10^9^/L or the platelet count was less than 100 × 10^9^/L.

### 2.7 Acute HT endpoints

The grading of HT adhered to the standardized criteria provided by the Common Terminology Criteria for Adverse Events (CTCAE v5.0) (23). Specifically, patients with a grade of 3 or higher for leukopenia, neutropenia, anemia, or thrombocytopenia within the focused RT duration were considered to have experienced acute HT. Moreover, in order to access the temporal progression of HT, the occurrence time of HT was also recorded and defined as between the initiation of radiotherapy and the point at which the patient exhibited the most severe HT symptoms within the focused RT duration (24).

### 2.8 Radiomics feature extraction

The original CT images were first resampled into a voxel size of [1 1 1]. After that, a total of 1221 radiomics features were extracted using Pyradiomics software (v3.1.0) (25) on treatment planning CT images for each of the defined three ROIs. The calculated radiomics features include the first-order features (e.g., image intensity), shape-based features (e.g., volume), and high-order features (e.g., texture features), which were simultaneously calculated in original images, wavelet filtered images, and Laplacian of Gaussian filtered images (see **Supplementary Tables 1, 2** for the list of all features). Specifically, the high-order feature sets contain four types of texture features, including the Gray-Level Co-occurrence Matrix (GLCM), Gray-Level Run Length Matrix (GLRLM), Gray-Level Size Zone Matrix (GLSZM), and Gray-Level Dependence Matrix (GLDM).

### 2.9 Feature selection and classification model for HT

In this discovery dataset, radiomics features (n = 1,221) of every ROI were combined with dosimetric (n = 51), demographic (n = 3), and clinical features (n = 4) together to construct the classification model for HT. Notably, models were generated for different cervical cancer treatment regimens: RT-alone group, CRT group, and their combination. The feature selection procedures were only conducted in the training dataset, and any information about the testing dataset was not leaked during the model training. Considering possible correlations between features, pairs of features with a correlation value greater than 0.90 were screened out, retaining the one with more significant between-group differences and discarding the other. A subsequent feature selection procedure was performed on the remaining radiomics features in the training dataset by conducting a two-sample t-test between patients with and without HT. Because no optimal p threshold can be confirmed at this moment, a set of p-values for the t-test was compared from 0.01 to 0.15 with an interval of 0.01, and the selected feature sets with the best classification performance on the testing dataset were reported. Finally, the widely used Support Vector Machine-Recursive Feature Elimination (SVM_RFE) (26, 31) with the linear kernel was adopted to determine the optimal feature sets on the training dataset, which was used to construct the classification model for HT. Of note, the strategy that used the optimal p threshold as the final model performance would not result in the data leak risk because every feature set was adopted for model construction, and the performance had been further validated with separate datasets.

According to the TRIPOD guidelines, the non-random split-sample development and validation were used in the discovery dataset, and another separate dataset was adopted for additional testing. In the discovery dataset, 80% of the total patients were selected as the training dataset with the remaining 20% patients for internal testing. Specifically, the training/testing samples were stratified to have a similar HT occurrence ratio. Considering the imbalance between positive and negative sample sizes, the synthetic minority over-sampling technique (SMOTE) was applied in the training process (32). Moreover, the validation dataset was used as a separate testing dataset to assess the model generalization. The model performance was evaluated according to the accuracy, sensitivity, specificity, and area under the curve (AUC). Every ROI formed a classification model for each group of the population, and the models were compared with each other to identify the most sensitive ROIs by DeLong’s test on AUC and representative feature sets. Furthermore, the models constructed with only radiomics features from BM were also constructed for comparison.

### 2.10 Time-to-event analysis for patients with HT

ROI displaying the best model classification was further used to study the temporal progression of HT using time-to-event analysis (33, 34). The model prediction probability of discriminative ROIs was used as HT risk indicators. The median of risk scores was applied to stratify patients into high- and low-risk groups illustrated by the Kaplan–Meier plot. Once the time-to-event model was significant, high-risk scores were assigned to patients with severe HT according to the log-rank test.

## 3 Results

**Table 2** summarizes the HT classification performance of three ROIs on the RT-alone group, CRT group, and combination group in the discovery dataset. The ROIs of BM achieved the best predictive performance in the three patient groups (Accuracy = 0.773, 0.778, and 0.729), and FH obtained the suboptimal results in the three groups (Accuracy = 0.750, 0.667, and 0.664). The CTV obtained relatively low HT classification accuracies (Accuracy = 0.591, 0.667, and 0.617). When only using radiomics features for model construction, the ROI of BM was selected for testing, and its performance was lower than that of the previous comprehensive model in the three groups (Accuracy = 0.682, 0.730, and 0.710). When the best models obtained in the discovery dataset were tested on the validation dataset, BM also exhibited the highest performance in three patient groups (Accuracy = 0.770, 0.839, and 0.854), while the FH consistently achieved the suboptimal performance (Accuracy = 0.770, 0.771, and 0.654), demonstrating the generalization, meaning the ability of a trained model to accurately make predictions on new, unseen data, of the HT classification model based on aforementioned two ROIs. However, the classification performance of CTV on validation datasets (Accuracy = 0.678, 0.619, and 0.624) was lower than that of the previous two ROIs.

**Table 2:** The classification model performance of different ROIs in discovery and validation datasets.

		RT alone			CRT			Combination		
ROI	Dataset	Accuracy	Sensitivity	Specificity	Accuracy	Sensitivity	Specificity	Accuracy	Sensitivity	Specificity
Comprehensive model										
BM	Discovery	0.773	0.818	0.636	0.778	0.861	0.600	0.729	0.740	0.706
	Validation	0.770	0.817	0.667	0.839	0.891	0.654	0.854	0.934	0.623
FH	Discovery	0.750	0.818	0.546	0.667	0.767	0.450	0.654	0.671	0.618
	Validation	0.770	0.833	0.630	0.771	0.870	0.423	0.654	0.704	0.509
CTV	Discovery	0.591	0.697	0.273	0.667	0.698	0.600	0.617	0.603	0.647
	Validation	0.678	0.733	0.556	0.619	0.608	0.654	0.624	0.645	0.566
Only radiomics model										
BM	Discovery	0.682	0.697	0.636	0.730	0.814	0.550	0.710	0.740	0.647
	Validation	0.646	0.676	0.538	0.757	0.800	0.577	0.731	0.733	0.722

The comprehensive model was generated using demographic, clinical, dosimetric, and radiomics features together.

BM, bone marrow; FH, femoral head; CTV, clinical target volume; RT, radiotherapy; CRT, chemoradiotherapy.

**Figure 2:**
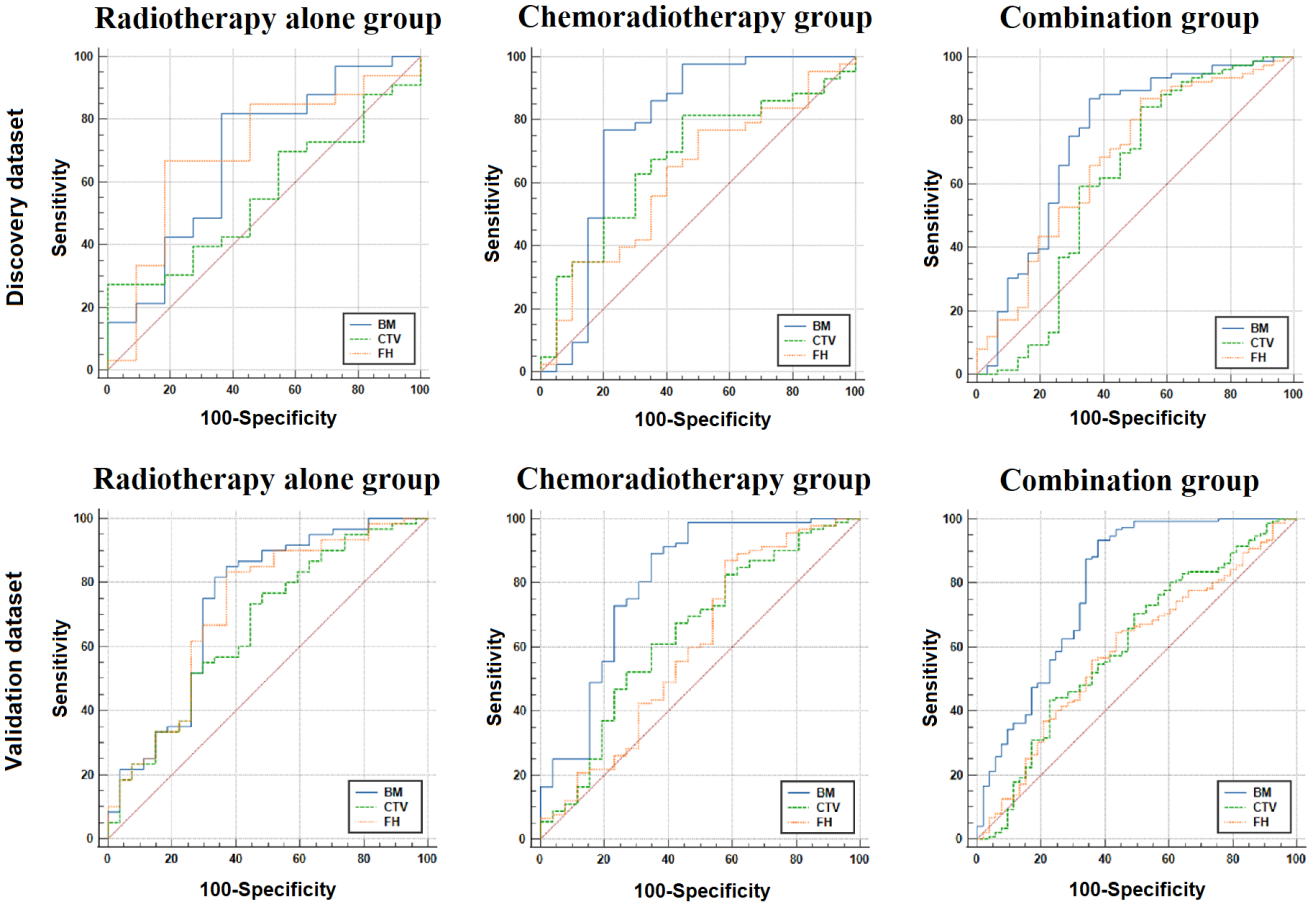
The ROC analysis for three ROIs in the discovery and validation dataset. BM, bone marrow; CTV, clinical target volume; FH, femoral head; ROC, receiver operating characteristic; ROIs, regions of interest.

In the discovery datasets, BM exhibited the highest AUC values of 0.686 (95% CI: 0.529–0.817), 0.779 (95% CI: 0.657–0.874), and 0.748 (95% CI: 0.655–0.827) for the RT-alone group, CRT group, and combination group, respectively. In the validation datasets, similar trends were observed for BM achieving superior performance with AUC values of 0.737 (95% CI: 0.612–0.862), 0.802 (95% CI: 0.6690–0.914), and 0.793 (95% CI: 0.713–0.874) for the three patient groups. The AUC values of FH and CTV were consistently lower than those of the BM, which are illustrated in **Figure 2** and **Table 3**. DeLong’s test revealed that there were significant differences (p < 0.05) in AUC between BM and CTV in the combination and RT-alone groups of the validation dataset as well as between BM and FH in the combination and CRT groups of the validation dataset.

**Table 3:** The comparison of AUC in different groups of discovery and validation datasets.

		RT alone	CRT	combination
ROI	Dataset	AUC (95% CI)	AUC (95% CI)	AUC (95% CI)
BM	Discovery	0.686 (0.529–0.817)	0.779 (0.657–0.874)	0.748 (0.655–0.827)
	Validation	0.737 (0.612–0.862)	0.802 (0.669–0.914)	0.793 (0.713–0.874)
FH	Discovery	0.697 (0.540–0.826)	0.621 (0.490–0.740)	0.615 (0.516–0.708)
	Validation	0.719 (0.594–0.843)	0.603 (0.4467–0.739)	0.590 (0.500–0.679)
CTV	Discovery	0.559 (0.402–0.708)	0.673 (0.543–0.786)	0.681 (0.584–0.768)
	Validation	0.663 (0.537–0.789)	0.642 (0.515–0.769)	0.607 (0.514–0.699)

BM, bone marrow; FH, femoral head; CTV, clinical target volume; RT, radiotherapy; CRT, chemoradiotherapy; AUC, area under the curve.

**Figure 3:**
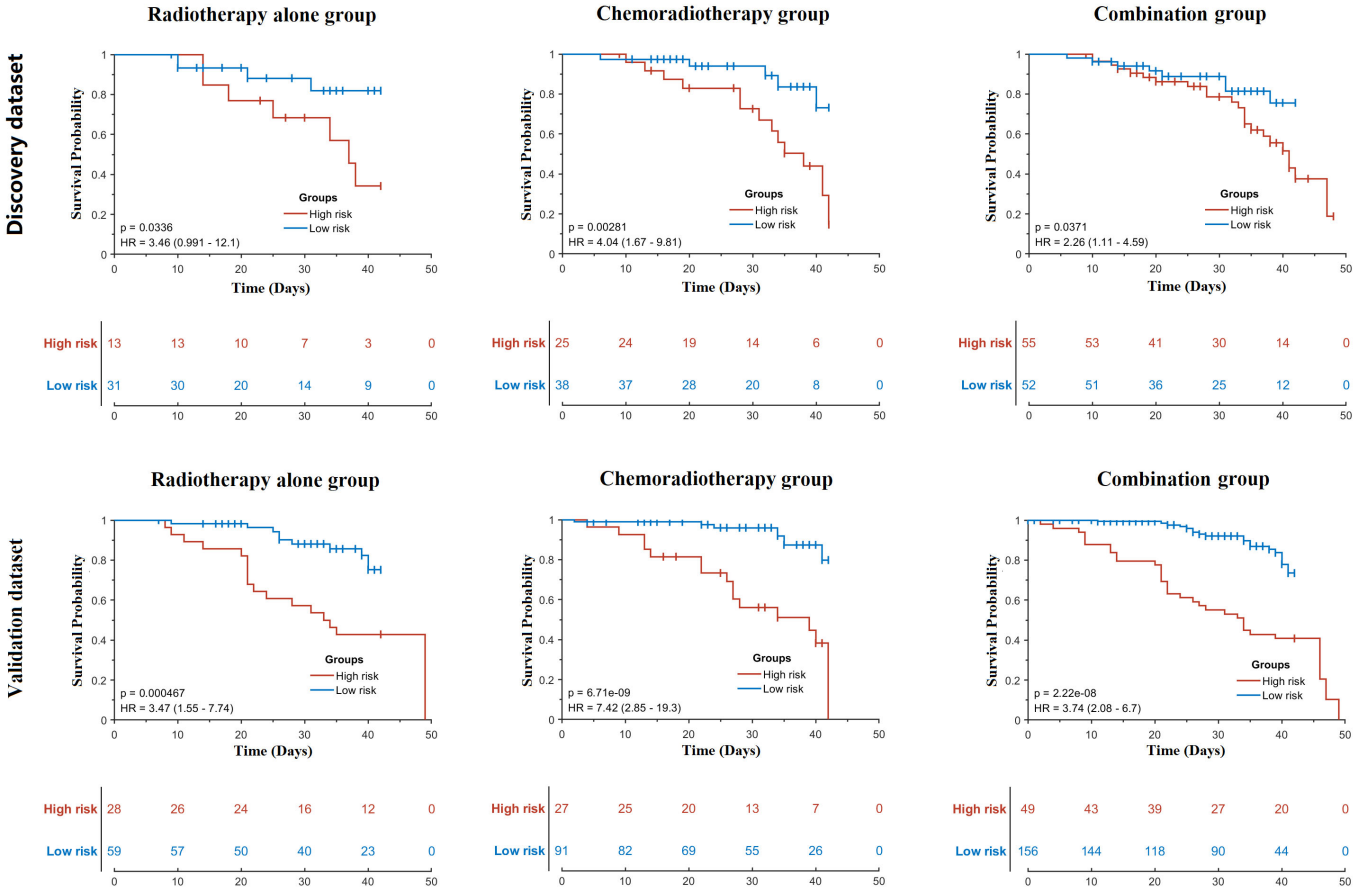
Time-to-event analysis of bone marrow for three patient groups in the discovery and validation datasets. p-Values were calculated using the log-rank test. HR, hazard ratio.

**Figure 4:**
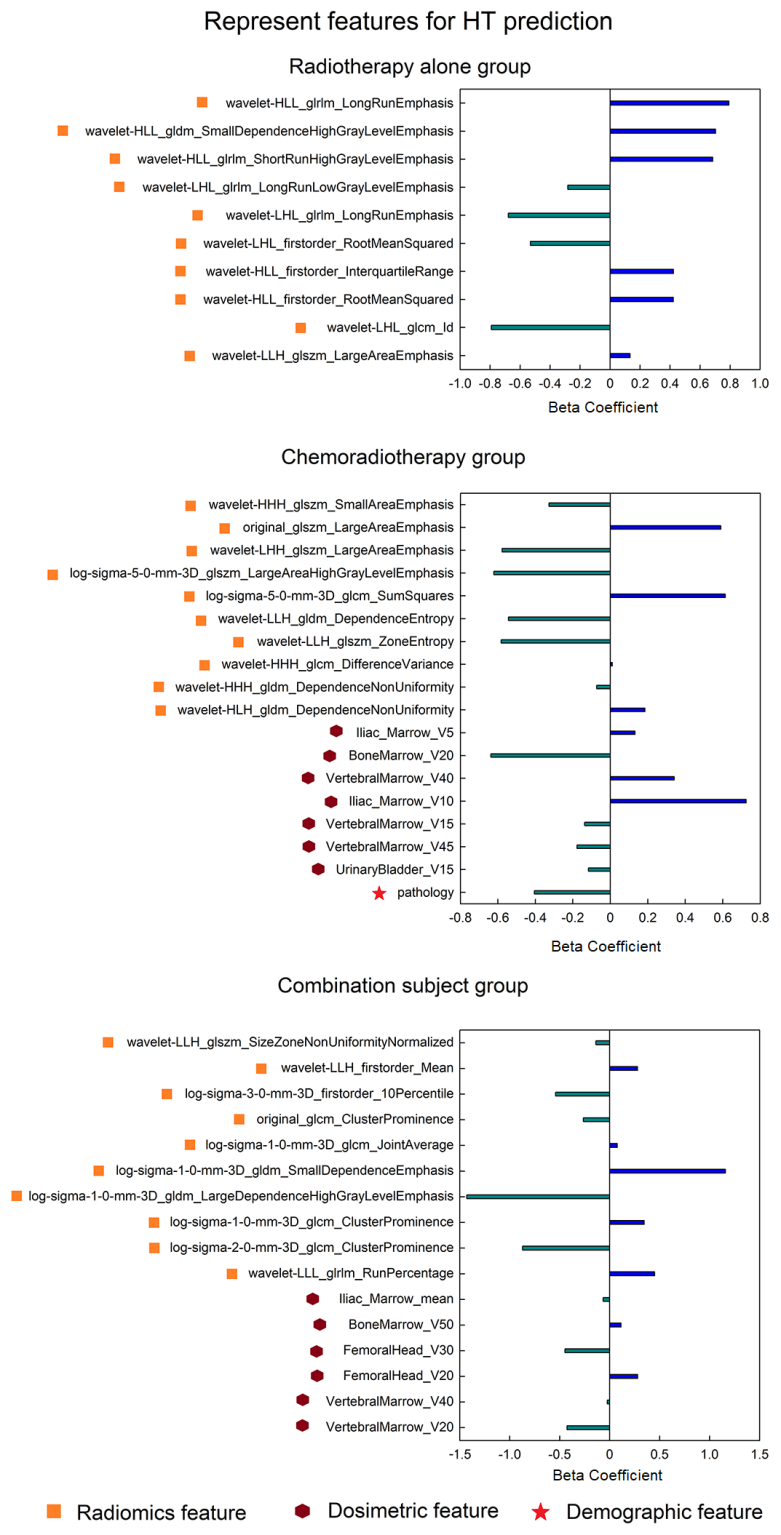
The most predictive feature sets (only displaying 10 representative radiomics features for each group) in the HT classification model for three patient groups. HT, hematologic toxicity.

In the time-to-event analysis, BM displayed significant log-rank test in three patient groups in both the discovery and validation datasets (**Figure 3**), indicating that BM is highly related to the occurrence time of HT. In addition, the hazard ratio for each group was also calculated, and all of them were larger than 3, implying that BM is a reliable risk factor for HT. For the optimal feature sets in the comprehensive model, different patient groups adopted distinct feature sets. In the RT-alone group, a total of 21 radiomics features (see **Supplementary Table 3**) were ultimately used but without any other types of features. In the CRT group, V5 and V10 of iliac marrow; V40, V45, and V15 of vertebral marrow; V20 of BM; and pathology were finally selected along with 104 radiomics features (see **Supplementary Table 4**). In the combination group, the mean dose of iliac marrow, V50 of BM, and V30 and V20 of FH, as well as V40 and V20 of vertebral marrow, were ultimately utilized along with other 36 radiomics features (see **Supplementary Table 5)**. To visually represent the feature sets in the classification model, the 10 most representative radiomics features together with discriminative dosimetric and demographic features are illustrated for three patient groups in **Figure 4**.

## 4 Discussion

In this study, we constructed HT prediction models for cervical cancer patients with comprehensive demographic, clinical, dosimetric, and radiomics features from three ROIs in different patient groups including the RT-alone group, CRT group, and combination group. The results revealed that BM was the most predictive ROI for HT prediction in all three groups compared to CTV and FH, which had been validated in both the discovery dataset and validation dataset. In addition, BM was also effective in stratifying the low-risk and high-risk patients during the HT progression. At last, distinct feature sets were adopted for different patient groups, and the dosimetric factors were related to HT occurrence, especially in the chemoradiotherapy patients.

Although several studies have reported the HT prediction using CT imaging radiomics features in LACC patients undergoing CRT, the study of Ren et al. (8) demonstrated that the integration of radiomics and clinicopathological parameters in a prediction model significantly enhanced the predictive capability for leukopenia in patients with LACC, resulting in an improved AUC of 0.699 compared to 0.564 and 0.551 achieved by clinical or radiological models alone. The study conducted by Le et al. (21) demonstrated that combining two clinical features (FIGO and postoperative chemotherapy cycles) with radiomics scores significantly improved the prediction of acute hematologic toxicity (grade ≥ 3) in LACC patients, achieving an impressive AUC of 0.88. This integrated model outperformed models utilizing either clinical or radiomics features alone. However, these studies did not selectively categorize subjects based on their treatment regimen. Our study first demonstrated that HT prediction in cervical cancer patients is dependent on the treatment regimen within the focused RT duration, and the diagnostic performance (**Figure 2** and **Table 3**) of the comprehensive model that combined demographic, clinical, dosimetric, and radiomics features was higher in the CRT patients than the RT-alone patients. Moreover, the results were attained using a large sample size and verified in the discovery and validation dataset. In addition, when two patient groups were combined together, the HT prediction model performed slightly lower than the model in the CRT patients but better than the model in the RT-alone patients. These results consistently indicate that the HT occurrence in the RT-alone group may be regulated by some other latent factors in addition to the radiomics and dosimetric factors [e.g., radiomics of 3D dose distribution (35, 36) or MRI-based radiomics (37, 38)], which needs to be investigated in the future.

Although the comprehensive model could effectively predict HT in cervical cancer patients, the results were dependent on the ROI choice. Our results revealed that BM is the most sensitive ROI than FH and CTV, while CTV performed the worst in all patient groups. Additionally, BM could also effectively predict the progressive endpoint for HT patients in the time-to-event analysis. Therefore, we infer that the occurrence of HT may be closely related to the status of BM prior the radiotherapy in cervical cancer patients. Furthermore, the performance of different ROIs in the validation dataset was similar to that in the discovery dataset, indicating the generalization of the ROI dependence in HT prediction.

The most representative features in the comprehensive model were also dependent on the patient groups. There were only 21 features adopted to construct the model for RT-alone patients, while there were respectively 111 and 42 features for the chemoradiotherapy and combination groups. In the RT group, no dosimetric factor was related to HT occurrence, and all features were radiomics features. This finding was consistent with our previous study on rectal cancer patients who underwent preoperative radiotherapy without any concurrent chemotherapy during the focused RT duration (39). Furthermore, the limited number of features may be a possible reason for the low model performance in the RT-alone group, although more selected features do not always lead to better model performance. In contrast, the CRT group showed substantial features as well as the best model performance. Moreover, the selected feature number in the model may be influenced by the feature selection manner; therefore, the final features may not be minimal for HT prediction. Specifically, there were more dosimetric factors in the CRT group than in the RT-alone group, indicating that the concurrent chemotherapy may make the patients more sensitive to the dosimetric strength. In comparison, in the previous two studies (8, 21) that predicted HT in cervical patients, there were no dosimetric features that have been selected in the final model. Additionally, pathology is also an indicator of HT occurrence in the CRT group, so we speculate the underlying mechanism of HT in the group may be complex based on the interaction of multiple factors. At last, the combination group adopted 42 features in all, and it is interesting that most of these features were different from those of either the RT-alone group or CRT group (feature overlap number = 3), again demonstrating the necessity of partitioning the whole cervical cancer patients into different treatment regimen groups.

There are several limitations in the study. First, the effective sample size in the study was not estimated, although it is within an acceptable range. In addition, all patients in the study may be influenced by the treatment interruption or blood transfusions, which will be explored in the future. Second, the neighborhood gray-tone difference matrix (NGTDM) features were not included in the study in order to simplify the subsequent analysis. Third, hormone therapy may have impacts on the bone marrow status, which may affect the model performance. Fourth, it is not easy to reveal the detailed explanation and clinical significance of every selected feature. At last, cross-validation may be more robust for uncovering the data heterogeneity than the non-random split-sample manner used in the study, which can be verified in future studies.

However, building an HT prediction model for real-world models is still challenging, and several factors may affect the model’s performance. For instance, the HT status can be promptly relieved, such as blood transfusions and administration of oral whitening medications (HT grade = 1) or consecutive 3-day injections of granulocyte colony-stimulating factor (HT grade = 2 or 3). However, it remains uncertain whether the constructed model would also exhibit efficacy in cases of recurrent HT. Second, there is still uncertainty regarding how administering chemotherapy 1 month prior to radiotherapy affects the occurrence of HT within the focused RT duration, highlighting the need for more refined grouping strategies in future studies. At last, future studies can use some advanced deep learning models to replace machine learning models on multi-center datasets, which may improve the model performance and generalization.

## 5 Conclusion

In the study, the comprehensive model that used demographic, clinical, dosimetric, and radiomics features together could effectively predict the HT within the RT duration in cervical cancer patients before radiotherapy, and BM is the most predictive ROI for HT occurrence and also useful in the detection of HT progression. Specifically, the model performances were obviously superior in the chemoradiotherapy patients than in the radiotherapy-alone patients.

## Data availability statement

The data analyzed in this study is subject to the following licenses/restrictions: The dataset couldn’t be shared with others currently. Requests to access these datasets should be directed to HY, yuehzh@163.com.

## Ethics statement

The studies involving humans were approved by Peking University Cancer Hospital & Institute. The studies were conducted in accordance with the local legislation and institutional requirements. The participants provided their written informed consent to participate in this study.

## Author contributions

HY: Conceptualization, Investigation, Software, Writing – original draft, Writing – review & editing, Formal analysis. XL: Writing – original draft, Writing – review & editing, Data curation, Resources, Validation. JY: Resources, Validation, Writing – original draft, Writing – review & editing. PF: Methodology, Software, Writing – original draft, Writing – review & editing. YD: Formal analysis, Software, Writing – original draft, Writing – review & editing. RW: Resources, Validation, Writing – original draft, Writing – review & editing. HW: Funding acquisition, Supervision, Writing – original draft, Writing – review & editing. JC: Resources, Supervision, Writing – original draft, Writing – review & editing. KD: Conceptualization, Resources, Supervision, Writing – original draft, Writing – review & editing. BJ: Conceptualization, Investigation, Methodology, Software, Writing – original draft, Writing – review & editing.
