# Supplementary material for: Acute hematologic toxicity prediction using dosimetric and radiomics features in patients with cervical cancer: does the treatment regimen matter?
Source: Front Oncol. 2024 May 21;14:1365897. doi: 10.3389/fonc.2024.1365897 (PMC11148289; doi:10.3389/fonc.2024.1365897)
Supplement: Supplementary file 1 [file DataSheet_1.docx]

**Supplementary information**

**Table S1: the shape features used in the study**

| The feature list only in original space |
| --- |
| original_shape_Elongation |
| original_shape_Flatness |
| original_shape_LeastAxisLength |
| original_shape_MajorAxisLength |
| original_shape_Maximum2DDiameterColumn |
| original_shape_Maximum2DDiameterRow |
| original_shape_Maximum2DDiameterSlice |
| original_shape_Maximum3DDiameter |
| original_shape_MeshVolume |
| original_shape_MinorAxisLength |
| original_shape_Sphericity |
| original_shape_SurfaceArea |
| original_shape_SurfaceVolumeRatio |
| original_shape_VoxelVolume |

**Table S2: the first-order and texture features used in the study**

| The feature list extracted from original space, Laplacian of Gaussian filter (1, 2, 3, 4, 5 mm) and wavelet (LLH、LHL、LHH、HLL、HLH、HHL、HHH、LLL) | |
| --- | --- |
| firstorder_10Percentile | glrlm_HighGrayLevelRunEmphasis |
| firstorder_90Percentile | glrlm_LongRunEmphasis |
| firstorder_Energy | glrlm_LongRunHighGrayLevelEmphasis |
| firstorder_Entropy | glrlm_LongRunLowGrayLevelEmphasis |
| firstorder_InterquartileRange | glrlm_LowGrayLevelRunEmphasis |
| firstorder_Kurtosis | glrlm_RunEntropy |
| firstorder_Maximum | glrlm_RunLengthNonUniformity |
| firstorder_MeanAbsoluteDeviation | glrlm_RunLengthNonUniformityNormalized |
| firstorder_Mean | glrlm_RunPercentage |
| firstorder_Median | glrlm_RunVariance |
| firstorder_Minimum | glrlm_ShortRunEmphasis |
| firstorder_Range | glrlm_ShortRunHighGrayLevelEmphasis |
| firstorder_RobustMeanAbsoluteDeviation | glrlm_ShortRunLowGrayLevelEmphasis |
| firstorder_RootMeanSquared | glszm_GrayLevelNonUniformity |
| firstorder_Skewness | glszm_GrayLevelNonUniformityNormalized |
| firstorder_TotalEnergy | glszm_GrayLevelVariance |
| firstorder_Uniformity | glszm_HighGrayLevelZoneEmphasis |
| firstorder_Variance | glszm_LargeAreaEmphasis |
| glcm_Autocorrelation | glszm_LargeAreaHighGrayLevelEmphasis |
| glcm_JointAverage | glszm_LargeAreaLowGrayLevelEmphasis |
| glcm_ClusterProminence | glszm_LowGrayLevelZoneEmphasis |
| glcm_ClusterShade | glszm_SizeZoneNonUniformity |
| glcm_ClusterTendency | glszm_SizeZoneNonUniformityNormalized |
| glcm_Contrast | glszm_SmallAreaEmphasis |
| glcm_Correlation | glszm_SmallAreaHighGrayLevelEmphasis |
| glcm_DifferenceAverage | glszm_SmallAreaLowGrayLevelEmphasis |
| glcm_DifferenceEntropy | glszm_ZoneEntropy |
| glcm_DifferenceVariance | glszm_ZonePercentage |
| glcm_JointEnergy | glszm_ZoneVariance |
| glcm_JointEntropy | gldm_DependenceEntropy |
| glcm_Imc1 | gldm_DependenceNonUniformity |
| glcm_Imc2 | gldm_DependenceNonUniformityNormalized |
| glcm_Idm | gldm_DependenceVariance |
| glcm_Idmn | gldm_GrayLevelNonUniformity |
| glcm_Id | gldm_GrayLevelVariance |
| glcm_Idn | gldm_HighGrayLevelEmphasis |
| glcm_InverseVariance | gldm_LargeDependenceEmphasis |
| glcm_MaximumProbability | gldm_LargeDependenceHighGrayLevelEmphasis |
| glcm_SumEntropy | gldm_LargeDependenceLowGrayLevelEmphasis |
| glcm_SumSquares | gldm_LowGrayLevelEmphasis |
| glrlm_GrayLevelNonUniformity | gldm_SmallDependenceEmphasis |
| glrlm_GrayLevelNonUniformityNormalized | gldm_SmallDependenceHighGrayLevelEmphasis |
| glrlm_GrayLevelVariance | gldm_SmallDependenceLowGrayLevelEmphasis |

**Table S3: all features used in the prediction model on radiotherapy alone patients**

| Feature list |
| --- |
| Rectum_mean |
| wavelet-HLL_glrlm_LongRunEmphasis |
| wavelet-HLL_gldm_SmallDependenceHighGrayLevelEmphasis |
| wavelet-HLL_glrlm_ShortRunHighGrayLevelEmphasis |
| wavelet-LHL_glrlm_LongRunLowGrayLevelEmphasis |
| wavelet-LHL_glrlm_LongRunEmphasis |
| wavelet-LHL_firstorder_RootMeanSquared |
| wavelet-HLL_firstorder_InterquartileRange |
| wavelet-HLL_firstorder_RootMeanSquared |
| wavelet-LHL_glcm_Id |
| wavelet-LLH_glszm_LargeAreaEmphasis |
| log-sigma-3-0-mm-3D_firstorder_RobustMeanAbsoluteDeviation |
| log-sigma-5-0-mm-3D_firstorder_10Percentile |
| log-sigma-5-0-mm-3D_firstorder_RootMeanSquared |
| log-sigma-5-0-mm-3D_glcm_ClusterProminence |
| wavelet-HLH_firstorder_RobustMeanAbsoluteDeviation |
| original_shape_MinorAxisLength |
| wavelet-HHL_firstorder_RootMeanSquared |
| wavelet-LHH_firstorder_RobustMeanAbsoluteDeviation |
| wavelet-HLH_glcm_ClusterProminence |
| log-sigma-4-0-mm-3D_glszm_SmallAreaLowGrayLevelEmphasis |
| log-sigma-2-0-mm-3D_glcm_Contrast |

**Table S4: all features used in the prediction model on chemoradiotherapy patients**

| Feature list |
| --- |
| wavelet-HHH_glszm_SmallAreaEmphasis |
| original_glszm_LargeAreaEmphasis |
| wavelet-LHH_glszm_LargeAreaEmphasis |
| log-sigma-5-0-mm-3D_glszm_LargeAreaHighGrayLevelEmphasis |
| log-sigma-5-0-mm-3D_glcm_SumSquares |
| wavelet-LLH_gldm_DependenceEntropy |
| wavelet-LLH_glszm_ZoneEntropy |
| wavelet-HHH_glcm_DifferenceVariance |
| wavelet-HHH_gldm_DependenceNonUniformity |
| wavelet-HLH_gldm_DependenceNonUniformity |
| wavelet-HLH_glcm_DifferenceVariance |
| wavelet-HLH_glcm_InverseVariance |
| wavelet-LHH_gldm_DependenceNonUniformity |
| wavelet-HHH_glcm_InverseVariance |
| log-sigma-3-0-mm-3D_gldm_DependenceNonUniformity |
| log-sigma-3-0-mm-3D_glcm_JointEntropy |
| log-sigma-2-0-mm-3D_glcm_JointEntropy |
| wavelet-LLH_gldm_DependenceNonUniformityNormalized |
| log-sigma-4-0-mm-3D_glszm_GrayLevelNonUniformity |
| log-sigma-4-0-mm-3D_gldm_SmallDependenceLowGrayLevelEmphasis |
| log-sigma-5-0-mm-3D_firstorder_Entropy |
| original_shape_SurfaceArea |
| wavelet-LLL_glrlm_ShortRunLowGrayLevelEmphasis |
| wavelet-HLH_glszm_ZoneEntropy |
| wavelet-HHH_gldm_LowGrayLevelEmphasis |
| wavelet-HHH_glszm_ZoneEntropy |
| wavelet-LHH_gldm_LowGrayLevelEmphasis |
| wavelet-LHH_glszm_ZoneEntropy |
| log-sigma-1-0-mm-3D_firstorder_InterquartileRange |
| log-sigma-1-0-mm-3D_glcm_Id |
| original_glcm_Id |
| wavelet-LHH_glcm_InverseVariance |
| log-sigma-5-0-mm-3D_glszm_GrayLevelNonUniformityNormalized |
| log-sigma-3-0-mm-3D_firstorder_Mean |
| wavelet-HLH_gldm_LowGrayLevelEmphasis |
| wavelet-HLH_firstorder_Mean |
| wavelet-HLH_firstorder_MeanAbsoluteDeviation |
| wavelet-HLH_glcm_ClusterProminence |
| wavelet-LHH_glcm_ClusterProminence |
| wavelet-LHH_firstorder_Mean |
| log-sigma-5-0-mm-3D_glrlm_LongRunHighGrayLevelEmphasis |
| original_shape_VoxelVolume |
| log-sigma-5-0-mm-3D_glcm_Contrast |
| log-sigma-4-0-mm-3D_glcm_Imc1 |
| wavelet-LHL_glrlm_RunEntropy |
| log-sigma-1-0-mm-3D_glrlm_RunEntropy |
| original_glrlm_ShortRunLowGrayLevelEmphasis |
| wavelet-LHL_glcm_Idm |
| log-sigma-2-0-mm-3D_glcm_Id |
| log-sigma-5-0-mm-3D_firstorder_Median |
| wavelet-LLH_glcm_ClusterProminence |
| log-sigma-4-0-mm-3D_glcm_Contrast |
| log-sigma-2-0-mm-3D_firstorder_Mean |
| log-sigma-1-0-mm-3D_gldm_SmallDependenceHighGrayLevelEmphasis |
| wavelet-LHH_glrlm_RunVariance |
| log-sigma-5-0-mm-3D_firstorder_10Percentile |
| log-sigma-4-0-mm-3D_firstorder_10Percentile |
| diagnostics_Mask-interpolated_Mean |
| log-sigma-1-0-mm-3D_firstorder_Kurtosis |
| log-sigma-1-0-mm-3D_firstorder_Minimum |
| wavelet-LHH_firstorder_RobustMeanAbsoluteDeviation |
| wavelet-HLL_gldm_SmallDependenceEmphasis |
| log-sigma-5-0-mm-3D_glszm_SmallAreaEmphasis |
| log-sigma-1-0-mm-3D_glcm_Contrast |
| log-sigma-2-0-mm-3D_gldm_SmallDependenceHighGrayLevelEmphasis |
| wavelet-HHH_glrlm_RunVariance |
| log-sigma-3-0-mm-3D_firstorder_RootMeanSquared |
| wavelet-HLH_glrlm_RunVariance |
| original_firstorder_Minimum |
| wavelet-HLL_glcm_ClusterProminence |
| log-sigma-1-0-mm-3D_firstorder_MeanAbsoluteDeviation |
| wavelet-LHL_firstorder_Kurtosis |
| log-sigma-2-0-mm-3D_firstorder_InterquartileRange |
| original_firstorder_InterquartileRange |
| wavelet-LHL_gldm_SmallDependenceHighGrayLevelEmphasis |
| log-sigma-2-0-mm-3D_glcm_Imc1 |
| log-sigma-1-0-mm-3D_firstorder_RootMeanSquared |
| log-sigma-2-0-mm-3D_firstorder_RootMeanSquared |
| log-sigma-5-0-mm-3D_glrlm_LowGrayLevelRunEmphasis |
| wavelet-LHL_firstorder_RootMeanSquared |
| log-sigma-2-0-mm-3D_glcm_Contrast |
| wavelet-LHL_firstorder_Minimum |
| wavelet-HHH_glszm_SmallAreaHighGrayLevelEmphasis |
| log-sigma-5-0-mm-3D_glcm_ClusterProminence |
| log-sigma-1-0-mm-3D_gldm_LargeDependenceEmphasis |
| original_gldm_SmallDependenceHighGrayLevelEmphasis |
| log-sigma-5-0-mm-3D_glszm_SizeZoneNonUniformityNormalized |
| wavelet-HHH_glcm_ClusterProminence |
| log-sigma-1-0-mm-3D_glszm_SmallAreaLowGrayLevelEmphasis |
| wavelet-HLL_gldm_LargeDependenceLowGrayLevelEmphasis |
| wavelet-LLH_firstorder_RootMeanSquared |
| Iliac_Marrow_V5 |
| wavelet-HHH_firstorder_Mean |
| log-sigma-5-0-mm-3D_firstorder_RootMeanSquared |
| original_glrlm_ShortRunHighGrayLevelEmphasis |
| original_firstorder_RootMeanSquared |
| original_shape_MinorAxisLength |
| wavelet-HLL_firstorder_RootMeanSquared |
| log-sigma-2-0-mm-3D_gldm_LargeDependenceEmphasis |
| BoneMarrow_V20 |
| wavelet-LHL_glszm_SmallAreaHighGrayLevelEmphasis |
| VertebralMarrow_V40 |
| log-sigma-1-0-mm-3D_glszm_SmallAreaHighGrayLevelEmphasis |
| Iliac_Marrow_V10 |
| VertebralMarrow_V15 |
| pathology |
| wavelet-LLH_glszm_SizeZoneNonUniformityNormalized |
| VertebralMarrow_V45 |
| wavelet-HHH_firstorder_RootMeanSquared |
| UrinaryBladder_V15 |
| wavelet-HHH_glrlm_GrayLevelNonUniformity |
| wavelet-HHH_glcm_SumEntropy |

**Table S5: all features used in the prediction model on combination patients**

| Feature list |
| --- |
| Iliac_Marrow_mean |
| wavelet-LLH_glszm_SizeZoneNonUniformityNormalized |
| wavelet-LLH_firstorder_Mean |
| Rectum_mean |
| Rectum_V5 |
| log-sigma-3-0-mm-3D_firstorder_10Percentile |
| original_glcm_ClusterProminence |
| log-sigma-1-0-mm-3D_glcm_JointAverage |
| log-sigma-1-0-mm-3D_gldm_SmallDependenceEmphasis |
| log-sigma-1-0-mm-3D_gldm_LargeDependenceHighGrayLevelEmphasis |
| log-sigma-1-0-mm-3D_glcm_ClusterProminence |
| log-sigma-2-0-mm-3D_glcm_ClusterProminence |
| wavelet-LLL_glrlm_RunPercentage |
| wavelet-LLH_glszm_SmallAreaLowGrayLevelEmphasis |
| log-sigma-1-0-mm-3D_glszm_GrayLevelVariance |
| wavelet-HLL_glrlm_ShortRunHighGrayLevelEmphasis |
| wavelet-HLL_glrlm_LongRunEmphasis |
| wavelet-HLL_glcm_Id |
| wavelet-HLL_gldm_SmallDependenceHighGrayLevelEmphasis |
| wavelet-HHL_firstorder_InterquartileRange |
| wavelet-HLL_firstorder_InterquartileRange |
| original_glszm_SmallAreaLowGrayLevelEmphasis |
| log-sigma-2-0-mm-3D_glcm_Imc1 |
| log-sigma-1-0-mm-3D_glszm_SmallAreaHighGrayLevelEmphasis |
| log-sigma-5-0-mm-3D_glcm_Contrast |
| original_shape_VoxelVolume |
| log-sigma-1-0-mm-3D_glszm_SmallAreaLowGrayLevelEmphasis |
| log-sigma-1-0-mm-3D_glcm_Contrast |
| BoneMarrow_V50 |
| FemoralHead_V30 |
| FemoralHead_V20 |
| wavelet-LLH_glszm_LargeAreaEmphasis |
| wavelet-HLL_firstorder_RootMeanSquared |
| wavelet-HHL_firstorder_RootMeanSquared |
| log-sigma-5-0-mm-3D_glszm_SizeZoneNonUniformityNormalized |
| wavelet-HLH_glcm_ClusterProminence |
| VertebralMarrow_V40 |
| VertebralMarrow_V20 |
| wavelet-HHL_glcm_Id |
| log-sigma-2-0-mm-3D_glcm_Contrast |
| original_shape_MinorAxisLength |
| wavelet-LLL_firstorder_90Percentile |
| wavelet-LLH_gldm_SmallDependenceHighGrayLevelEmphasis |
| wavelet-LLH_glszm_SmallAreaHighGrayLevelEmphasis |
